# Burst swimming in sockeye salmon during their 1100 km upstream migration

**DOI:** 10.1093/conphys/coag066

**Published:** 2026-09-12

**Authors:** Kim Birnie-Gauvin, Kendra A Robinson, Evan E Byrnes, Jacey C Van Wert, Jeremy G Venditti, Steven J Cooke, Erika J Eliason, David A Patterson

**Affiliations:** Department of Ecology, Evolution and Marine Biology, University of California Santa Barbara, Santa Barbara, CA 93106, USA; Section for Freshwater Fisheries and Ecology, Technical University of Denmark, Vejlsøvej 39, 8600 Silkeborg, Denmark; Department of Fisheries and Oceans, Aquatic Research Cooperative Institute, School of Resource and Environmental Management, Simon Fraser University, Burnaby, BC V5A 1S6, Canada; School of Environmental Science, Simon Fraser University, Burnaby, BC V5A 1S6, Canada; Oceans Department, Doerr School of Sustainability, Hopkins Marine Station, Stanford University, Pacific Grove, CA 93950, USA; Section for Freshwater Fisheries and Ecology, Technical University of Denmark, Vejlsøvej 39, 8600 Silkeborg, Denmark; School of Environmental Science, Simon Fraser University, Burnaby, BC V5A 1S6, Canada; Fish Ecology and Conservation Physiology Laboratory, Department of Biology and Institute of Environmental and Interdisciplinary Science, Carleton University, 1125 Colonel By Drive, Ottawa, ON K1S 5B6, Canada; Section for Freshwater Fisheries and Ecology, Technical University of Denmark, Vejlsøvej 39, 8600 Silkeborg, Denmark; Pacific Science Enterprise Centre, Fisheries and Oceans Canada, West Vancouver, BC V7V 1H2, Canada; Department of Fisheries and Oceans, Aquatic Research Cooperative Institute, School of Resource and Environmental Management, Simon Fraser University, Burnaby, BC V5A 1S6, Canada

**Keywords:** Accelerometer, aerobic, anaerobic, biologging, exercise physiology, Fraser River, locomotion, Pacific salmon

## Abstract

In the Fraser River (British Columbia, Canada), some sockeye salmon (*Oncorhynchus nerka*) populations migrate upwards of 1100 km to reach their spawning grounds and encounter challenging river conditions. Understanding how sockeye salmon partition their finite energy budget into aerobic (sustained) and anaerobic (burst) swimming could help us understand why some fish successfully make it to the spawning grounds while others do not. We tagged sockeye salmon upon their entry in freshwater with archival accelerometer radio tags and recovered 16 tags on the spawning grounds, providing us with the first full migration datasets from which we can quantify burst swimming. We found that the type of bursts (e.g. duration, maxima, peak count and effort) varies along their migration, likely reflecting changing flow dynamics and channel morphology, but that on average, fish only spend 1.2% of their migration bursting. Unsurprisingly, the Fraser Canyon required significantly more bursting than the remainder of their migration, representing 75.3% of the cumulative bursting effort despite representing only 35% of the distance. In this reach, sockeye salmon performed on average 4.5 bursts per km and 117 bursts per day, representing an effort of 40.0 *g* km^−1^, which is significantly more than in any other reach. Moreover, we found that in the early phases of the migration (Lower Fraser and Canyon), salmon avoided bursting at night-time. We did not however find evidence of individual repeatability in the types of bursts that individual fish perform. Our study is the first to quantify burst swimming over a 1100 km migratory route in sockeye salmon. Our findings indicate that despite the repeated need to burst, salmon are equipped to do so in an energy-conserving way. This knowledge is useful for resource managers as they make decisions about mitigation actions that could make migrations easier especially in the context of a warmer future.

## Introduction

Energy is the currency of life (Kleiber, 1961), and how animals partition this energy into different functions and activities poses an important trade-off that must be balanced (Soofiani and Hawkins, 1985; Stearns, 2000). This is particularly salient to animal migrations that are often arduous and require extensive endogenous resources to fuel movement (Dingle and Drake, 2007). Energetic trade-offs for migratory animals are of critical importance now more than ever before as animals face increasingly intense challenges, such as habitat fragmentation (Cooke *et al.*, 2024) and climate change (Robinson *et al.*, 2009). In fishes, the cost of activity (i.e. swimming) typically represents the largest and most variable component of their energy budget (Boisclair and Leggett, 1989; Boisclair and Sirois, 1993) and, as such, is generally the first to be adjusted in response to energetic constraints. Understanding swimming performance and how it is affected by internal (i.e. physiology) and external (i.e. environment) factors is fundamental to predicting how fish are likely to cope with environmental change and for focusing conservation efforts aimed at mitigating anthropogenic impacts (Metcalfe *et al.*, 2016). However, estimating energy use is challenging in aquatic organisms, particularly in the wild (Chipps and Wahl, 2008).

Swim flumes have been used for decades to estimate energy use because swimming speed and tail-beat frequency (TBF) closely correlate with oxygen consumption (e.g. Brett, 1995; Hinch and Rand, 1998; Brown *et al.*, 2007; Payne *et al.*, 2011). More recently, improvements in biotelemetry and biologging technology have greatly improved our ability to estimate energy use in fish (Cooke *et al.*, 2016), especially with the advent of animal-borne acceleration tags. This is because locomotion results from muscle contractions that lead to body acceleration, making acceleration a reliable proxy for energy expenditure (Gleiss *et al.*, 2011) across taxa (Wilson *et al.*, 2020). In fishes, acceleration data have been used to estimate energy use during homing and spawning in salmon (Tanaka *et al.*, 2001; Tsuda *et al.*, 2006) and steelhead trout (*Oncorhynchus mykiss*; Fuchs and Caudill, 2019), diel activity patterns in sharks (Whitney *et al.*, 2007) and bonefish (*Albula vulpes*; Murchie *et al.*, 2011), and sockeye salmon ascending fishways (*Oncorhynchus nerka*; Burnett *et al.*, 2014). Such data are particularly useful in supporting resource management and conservation decisions because they capture fine-scale behaviour continuously over space and time, leaving little to no gaps in the observations of individual animals thus reducing uncertainty.

Somatic energy reserves are an important and limited commodity, particularly for upstream migrating adult Pacific salmon (*Oncorhynchus* spp.). These fish cease feeding prior to their entry into freshwater from the ocean and can migrate hundreds of kilometres before reaching their spawning grounds for a single opportunity to reproduce (i.e. they are semelparous capital breeders). The upriver migration, gonadal growth and development of secondary sexual characteristics, and spawning behaviours are all fuelled by these endogenous energy stores. In the Fraser River (British Columbia, Canada), adult salmon must also overcome multiple areas of difficult passage, including the infamous Hell’s Gate and the more recent Big Bar Landslide (Ricker, 1947; Hinch and Bratty, 2000; Wright *et al.*, 2024). Some populations, like the Early Stuart and Nadina sockeye salmon that are the focus of this paper, face an even more challenging migration as their earlier migration timing coincides with the end of spring freshet. In fact, these populations have been assessed as endangered (Nadina) and critically endangered (Early Stuart) in part due to the high in-river mortality (COSEWIC, 2021).

As they ascend the Fraser River, sockeye salmon must burst swim—brief, high-intensity swimming bouts. Sockeye salmon begin their freshwater migration in the Lower Fraser River, where the river is considered alluvial, and where flows that would challenge a salmon’s maximum swimming ability are not produced (Venditti and Church, 2014; Kraskura *et al.*, 2024). In this reach, salmon may still need to burst swim to escape predators like seals, but river morphology and flow dynamics are not considered a major challenge. In the 375 km long Fraser Canyon, upstream of the Lower Fraser, the river is bedrock-bound (rock on both banks) for ~25% and constricted by rock on one bank for another 25% of the length of the canyon (Rennie *et al.*, 2018; Wright *et al.*, 2022). The rock cannot be easily eroded and can produce flows that can challenge or exceed the swimming capabilities of salmon. In many bedrock-bound reaches, salmon must therefore burst swim to overcome hydraulic challenges (Standen *et al.*, 2002). Moreover, new landslides can occur and create new hydraulic challenges for salmon; such was the case for the Big Bar Landslide, which occurred in late 2018 and completely blocked salmon migration in the early summer of 2019. In the Upper Fraser, upstream of the Fraser Canyon, salmon face significantly less challenging flow dynamics than in the Canyon. Early Stuart and Nadina sockeye salmon navigate two lakes before reaching their spawning creeks. Little is known about their lake passage, though we do not expect that bursting would represent a significant component of their swimming here. On the spawning grounds, sockeye salmon may burst swim due to low water flow, increased spawning activity and likely to escape predators like bears (Healey *et al.*, 2003).

Bursting relies on anaerobic metabolism, which is less efficient and more costly than aerobic metabolism (Hochachka and Somero, 2002), and requires time for recovery (Birnie-Gauvin *et al.*, 2023). However, how much and how hard sockeye salmon have to burst throughout their entire freshwater migration to their spawning grounds remains unknown. To date, few studies have explored burst swimming in free-swimming salmon, and those that have done so have tracked animals over short durations or at specific locations (e.g. Hinch and Rand, 1998; Hinch and Bratty, 2000; Fuchs and Caudill, 2019). Even so, those studies were unable to describe the types of bursts performed (e.g. duration and effort). Given the need for sockeye salmon to perform repeat burst swimming over several weeks until they reach their spawning grounds, we aimed to quantify anaerobic burst swimming throughout their entire migration, from shortly after river entry to their spawning grounds. Salmon experience different challenges along their migration and thus likely burst for different reasons in different parts of the river system (Lower Fraser, Fraser Canyon, Upper Fraser, and lakes and spawning creeks), so we expected that the quantity and types of bursts performed would differ across these reaches.

We used animal-borne jerk acceleration loggers to quantify burst swimming activity throughout the entire ~1100 km freshwater migration of endangered Early Stuart and Nadina sockeye salmon, characterizing when, where and how intensely fish engaged in energetically costly anaerobic swimming. Our data are the first of their kind in that they provide unprecedented insight into the quantity and type of bursting that sockeye salmon perform across their full upstream migration. Repeated anaerobic activity is costly, prolonging recovery, depleting energy reserves, delaying migration and impairing predator-avoidance behaviours (Eliason and Farrell, 2016). Therefore, understanding sockeye salmon swim performance is necessary for assessing the factors contributing to mortality during their migration. This research can help identify areas that pose significant challenges for these fish which is of use to resource managers and conservation decision-makers tasked with undertaking habitat enhancement or restoration efforts that may be necessary to assist migrating salmon in a warmer world or after events such as landslides.

## Materials and methods

### Study system

The Fraser River drains 232 000 km^2^ of central and south-western British Columbia (Canada; Rennie *et al.*, 2018). The main stem of the river is ~1375 km and the river flows from the Rocky Mountains, across the Interior Plateau, between the Coast and Cascade Mountain ranges and then to the Pacific Ocean. Flow in the Fraser River is snow-melt-dominated, with high flows in the spring and early summer, and low flows through the fall and winter. At the exit of the Fraser Canyon, the mean annual peak flow is 8420 m^3^ s^−1^, the mean annual flow is 2700 m^3^ s^−1^ and the base flow is ~1000 m^3^ s^−1^ (Hurson *et al.*, 2025).

The river is home to many populations of Pacific salmon, including the Early Stuart and Nadina sockeye salmon, which spawn in the small creeks around Trembleur and Takla Lakes, and the Nadina River, respectively. Both populations migrate ~800 km in the mainstream of the Fraser River, passing through the Fraser Canyon, before entering the Nechako River. Their migration diverges 92 km later, where Early Stuart sockeye salmon turn north into the Stuart River. The complete migration from river entry to the start of the spawning grounds is 1085 and 1133 km for Early Stuart and Nadina sockeye salmon, respectively. In the Fraser River, sockeye salmon typically migrate upstream starting in early July and ending in October. The Early Stuart and Nadina sockeye salmon populations typically begin their migration in early July and, as such, are among the first sockeye salmon populations to begin their ascent.

For this study, we separated the Fraser River into four reaches, based on river channel morphology, and the presence of radio antennas to detect fish as they progressed upstream. The Lower Fraser (release to Qualark, RK 77–RK 184, 107 km) is characterized as an alluvial river channel with sand and gravel beds. Next is the Fraser Canyon (Qualark to Soda, RK 184–RK 569, 385 km), which is characterized as an intermittent bedrock channel, with some narrow sections associated with high velocity plunging flows (Venditti *et al.*, 2020). Note that the true start of the Fraser Canyon is at RK 190, not RK 184, but the positioning of our radio antennas was at RK 184. The next reach, the Upper Fraser (Nadina: Soda to Nautley, RK 569–RK 991, 422 km; Early Stuart: Soda to Stuart, RK 569–RK 994, 425 km), is predominately alluvial with a few bedrock sections, though with no major hydraulic challenge. The last reach is the lakes and spawning grounds (Nadina: Nautley to spawning grounds, RK 991–RK 1133, 142 km; Early Stuart: Stuart to spawning grounds, RK 994–RK 1085, 91 km). We were unable to accurately distinguish between the lakes and spawning creeks given the lack of tracking data in these remote places. Little is known about lake passage, but they are not considered challenging for the fish. The spawning creeks do not necessarily represent hydraulic challenges, though low flows, bears, beaver dams and spawning activity are expected to require burst swimming.

### Fish capture and tagging

**Figure 1:**
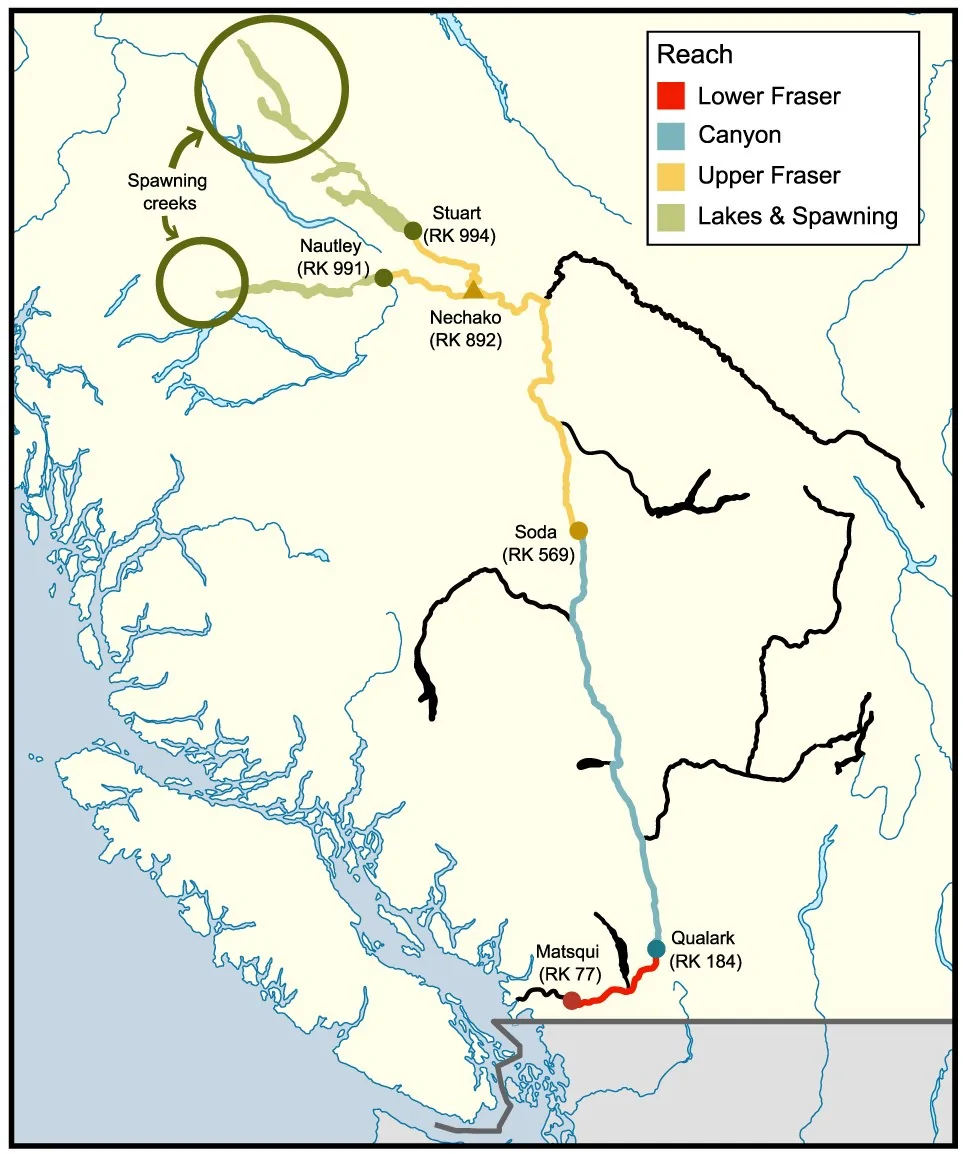
Map of the Fraser River watershed. Coloured river reaches correspond to the river reaches used in the analysis, with red indicating the Lower Fraser River from Matsqui to Qualark (107 km), blue indicating the Fraser Canyon (385 km), yellow indicating the Upper Fraser River (Early Stuart: 425 km; Nadina: 422 km) and green indicating the lakes and spawning grounds (Early Stuart: 91 km; Nadina: 142 km). Tagging and release location (Matsqui) at RK 77 is indicated by a red dot. All other coloured shapes were equipped with radio antennas; detections at these antennas were used to track when individuals were within each river reach. Where Early Stuart sockeye salmon turn north from the Nechako River is indicated by a yellow triangle. Lake entry is indicated by the green dots. Green circles indicate the area with spawning creeks, past the lakes.

Sockeye salmon were captured and tagged on 13 and 15 July 2021 (30 individuals) and on 12 and 26 July 2022 (30 individuals) in the Lower Fraser River, near Matsqui, British Columbia (Fig. 1). Fish were captured using a fishwheel (Robichaud *et al.*, 2008; Cook *et al.*, 2014). Upon capture, the fish would slide into livewells on either side of the wheel, where fresh river water flowed through. Fish were netted from these livewells and moved into a partly submerged trough to be measured, sampled and tagged. Fish were selected based on their physical condition (i.e. no fungus or large wounds) and energetic status (i.e. high fat content), as determined using a fish fat probe (model FFM-692 on sockeye salmon setting; Distell Inc., West Lothian, Scotland; see Crossin and Hinch, 2005) to select individuals most likely to survive and reach their spawning grounds, given that this was the objective of the study. A small piece of adipose tissue was collected for population identification (see Beacham *et al.*, 2005). A 1.5 ml blood sample was taken from the caudal vasculature for a separate study. Finally, fish were gastrically tagged with ID and acceleration radio-archival tags (‘jerk’ tags, MCFT3-3A, Lotek Wireless Inc., Newmarket, Ontario, Canada). Fish were then released into the river at their site of capture. This protocol was approved by Simon Fraser University Animal Care Committee 1339E-22.

Flow and temperature conditions in the Fraser River differed between the two study years (2021 and 2022). Peak flow between 12 July and 16 September (first and last dates of dataset) was 5060 m s^−3^ in 2021 and 9030 m s^−3^ in 2022, while temperature was slightly elevated in 2021 compared to 2022 (Fig. S1).

### Tag data and tracking

The tags transmitted unique ID and jerk values (*g* s^−1^, where *g* is gravity and equals 9.8 m s^−2^) every 2 s and archived jerk values every 6 s at a sampling rate of 50 Hz. The jerk values used here were the vectorial sum of jerk, which is calculated as the vectorial sum of the mean jerk values from all three axes (Van Wert *et al.*, 2026). The mean jerk values from each axis are calculated as the difference between consecutive observations and averaged across the entire sampling window (2 and 6 s respectively for transmitted and archived data). Note that jerk is defined as the rate of change in acceleration (m s^−3^) and therefore is not equivalent to most acceleration data. Jerk values ranged from 0 to 2 *g* s^−1^, with values of zero representing no change in acceleration, thus reflecting consistent swimming. The estimated battery life for the tag was 237 days.

Telemetry arrays consisted of Orion receivers (Sigma Eight Inc., Newmarket, ON, Canada) with radio antennas (Yagi type, three or four elements) positioned throughout the Fraser River in gates, detecting tagged fish as they swam by (see Fig. 1). This enabled us to associate archived jerk values (see below) to specific river reaches.

### Tag recovery

The spawning grounds of the two populations of focus were walked (2021: Early Stuarts; 2022: Early Stuarts and Nadina) with a hand-held radio receiver and an antenna to recover tagged fish that had completed their upstream migration. In 2021, three tags were recovered from Early Stuart sockeye salmon (all males). In 2022, 13 tags were recovered: three from Early Stuart (two males and one female) and 10 from Nadina sockeye salmon (five males and five females). The recovery of tags on the spawning grounds was critical as this is what gave us access to the full migration dataset, with a 6 s resolution, rather than relying on transmitted data where antennas were present.

### Bursting: lab calibration and tag data

**Figure 2:**
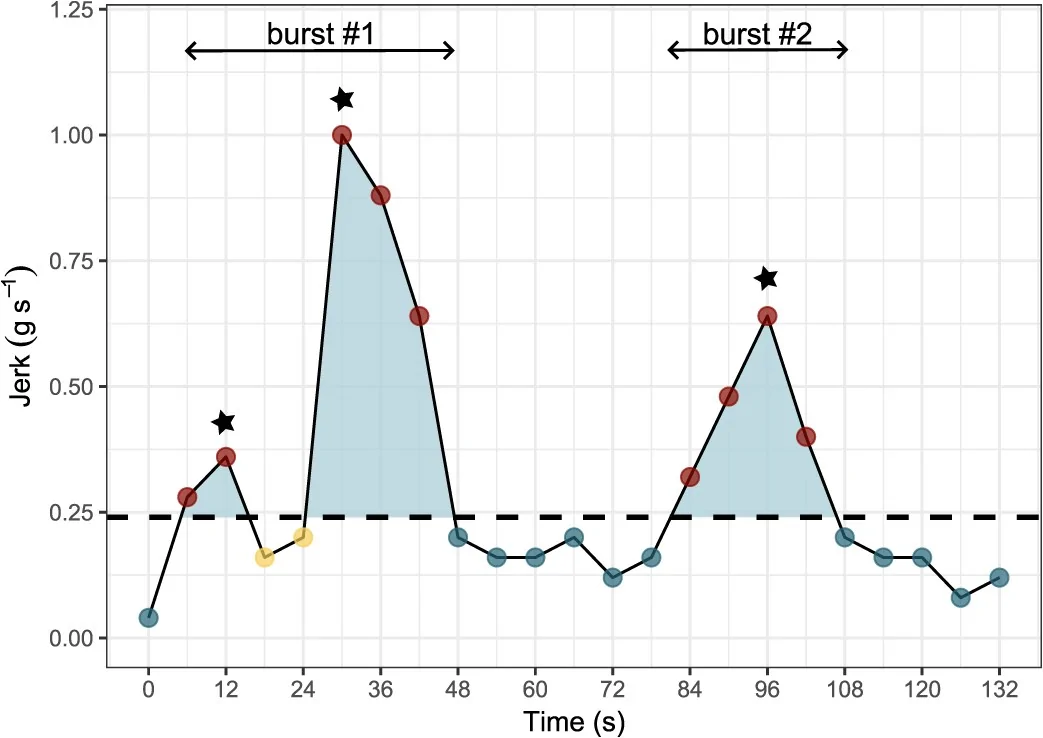
Schematic of burst definition and burst metrics. A burst event is considered any event when jerk is greater than the threshold (dashed horizontal line), here represented as red points. If jerk values dropped below the threshold for 30 s or less (yellow points) before going back above the threshold, this was considered as a single burst event (Burst #1). If jerk values dropped below the threshold for >30 s (blue dots) before going back above the threshold, this was considered a new burst event (Burst #2). The duration of each burst is represented by the double-headed horizontal arrows; stars represent peaks. Burst #1 has two peaks, a max amplitude of 1.0 and a duration of 36 s. Burst #2 has one peak, a max amplitude of 0.625 and a duration of 18 s. The shaded area under the curve represents burst effort.

Our primary interest with the archived data from the recovered tags was to quantify and characterize bursting (i.e. anaerobic swimming) during the upstream migration. To determine the jerk threshold corresponding to anaerobic swimming, we performed a laboratory calibration study using a swim flume (see Section 2 of Supplementary  Materials for details; Figs S7, S8 and S9). In brief, sockeye salmon caught early in the upstream migration were brought to the laboratory at Simon Fraser University and tagged with both Axy 5S high resolution acceleration tags (Technosmart, Italy) and the ‘jerk’ tags (Lotek, Canada). The fish were then placed in a 10 m swim flume (see Fig. S7) and forced to swim from sustained swimming to burst-and-coast swimming to burst swimming, until fatigue (Beamish, 1978). We first evaluated the relationship between jerk and TBF using triaxial accelerometers and subsequently related TBF to swimming speed using published work on the transition from aerobic to anaerobic fuelling to ultimately establish a jerk threshold for burst swimming (for more details and images, see Section 2 of Supplementary Materials). From this work, we determined that the jerk threshold for bursting was ~0.24 *g* s^−1^. As such, we defined burst events as any event when jerk was > 0.24 *g* s^−1^. We allowed up to 30 consecutive seconds of jerk values below the threshold within a burst event to consider burst-and-coast swimming as well (Fig. 2). This means that if jerk values dropped below the threshold for >30 s, then the next jerk value above the threshold was considered a new burst event. Notably, we attempted different durations for these ‘drops below the threshold’ and found that this duration led to the most representative bursts. See Fig. S2 for representative examples of bursts.

Raw jerk records were inspected for temporal gaps prior to analysis. Gaps were present in all datasets, representing ~1.6% of each record; the majority (98.7%) were exactly 12 s in duration, indicating a single missed logged value, with the remainder spanning 12–72 s. Because our burst event detection algorithm tolerates sub-threshold intervals of up to 30 s, gaps of this magnitude are unlikely to affect burst identification and no imputation was performed.

### Analyses

Where, when and how sockeye salmon burst: from each burst event, we extracted four characteristics: duration (seconds), maximum amplitude (peak jerk value), cumulative burst effort (area under the jerk curve; *g*) and number of peaks (local maxima where jerk values exceeded both the preceding and following data points) (Fig. 2). We also calculated the refractory period (time between burst events). These metrics were then used to characterize the types of bursts performed (e.g. long with high effort; short with low effort) across four reaches along the Fraser River.

We compared burst types across the four identified reaches using generalized linear mixed models (GLMMs; *glmmTMB*, Brooks *et al.*, 2017) with river reach as predictor and fish ID as a random factor (given the thousands of bursts per individual), and either maximum amplitude, duration, effort, peak count or refractory period as the response variable. Because all burst characteristics data were left skewed, we rescaled the data between zero and one. Burst amplitude data were bound (minimum = 0.24, maximum = 2.0), so the data were analysed using a beta GLMM. Duration, effort and refractory period data were analysed using a gamma GLMM, whereas peak count data were analysed using a negative binomial GLMM. We also tested whether the occurrence of burst events was affected by hour of day (0–23) using a negative binomial GLMM for count data, with river reach as a predictor and fish ID as a random factor.

Individual differences: we investigated whether there was strong interindividual variation in bursting characteristics given that each individual performed 2014 bursts or more. To do so, we used the same approach as described in the previous section, using GLMMs with fish ID as a random factor and reach as a predictor. We then calculated repeatability (intraclass correlation coefficients, ICC) in burst traits within individuals (performance, Lüdecke *et al.*, 2021). Previous research had documented reasonable repeatability of overall travel speed among reaches (Hanson *et al.*, 2008), yet no studies have assessed repeatability in busting activity. Here, ICC values of 0.5 or higher were considered moderately repeatable/specialized (Koo and Li, 2016). All analyses were conducted in R (version 4.3.2).

## Results

**Figure 3:**
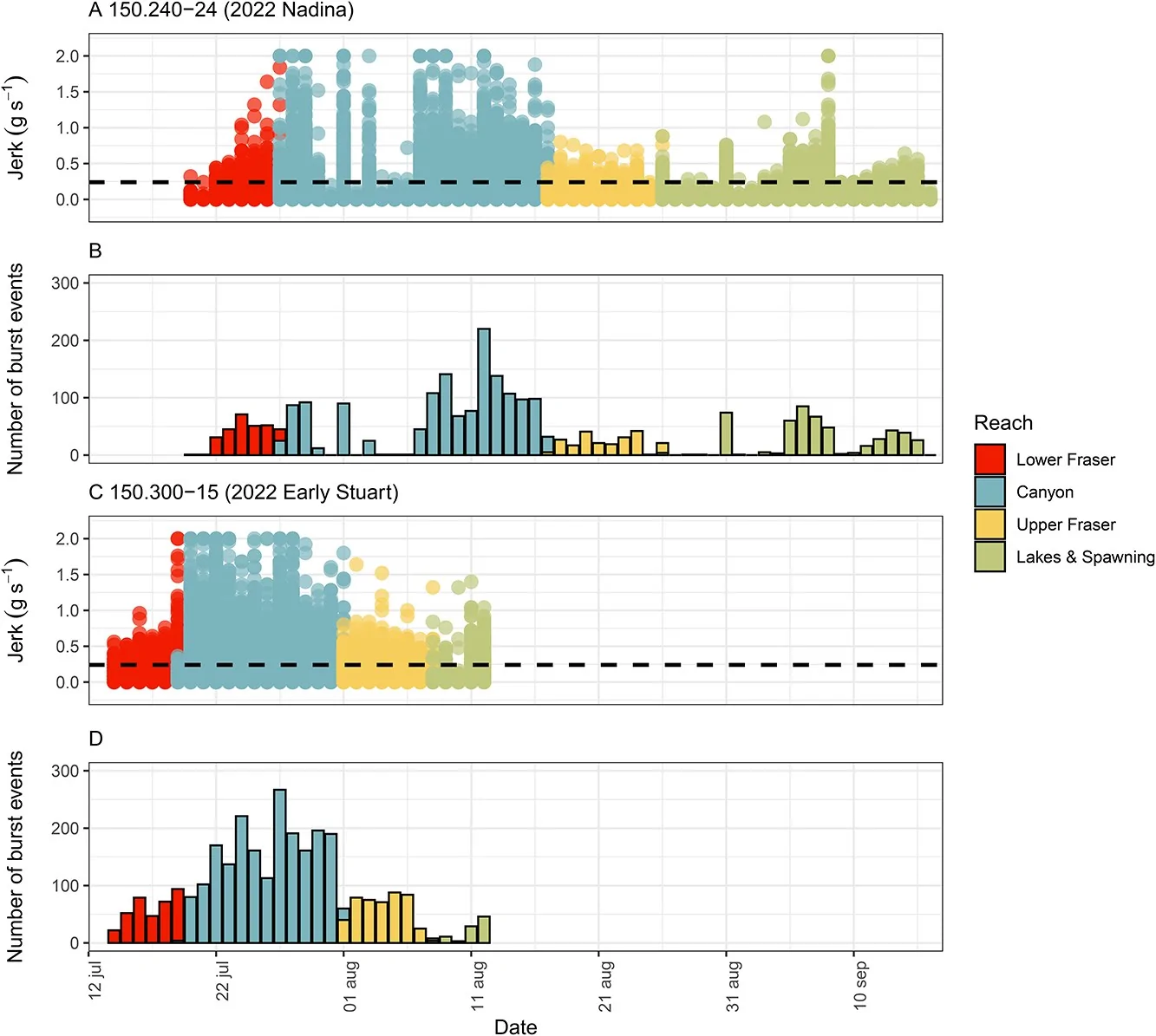
The migration of sockeye salmon in terms of anaerobic burst swimming at a daily time step. Raw jerk (*g* s^−1^) and number of burst events per day during the migration of a representative Nadina (A, B) and Early Stuart (C, D) sockeye salmon, coloured by reach. The dashed horizontal line at 0.24 indicates the bursting threshold. Note that dates are only labelled every 10 days, but all graphs start on 12 July and end on 17 September. Similar plots for the remaining 12 individuals from this study can be found in Supplementary Materials (Fig. S3).

**Figure 4:**
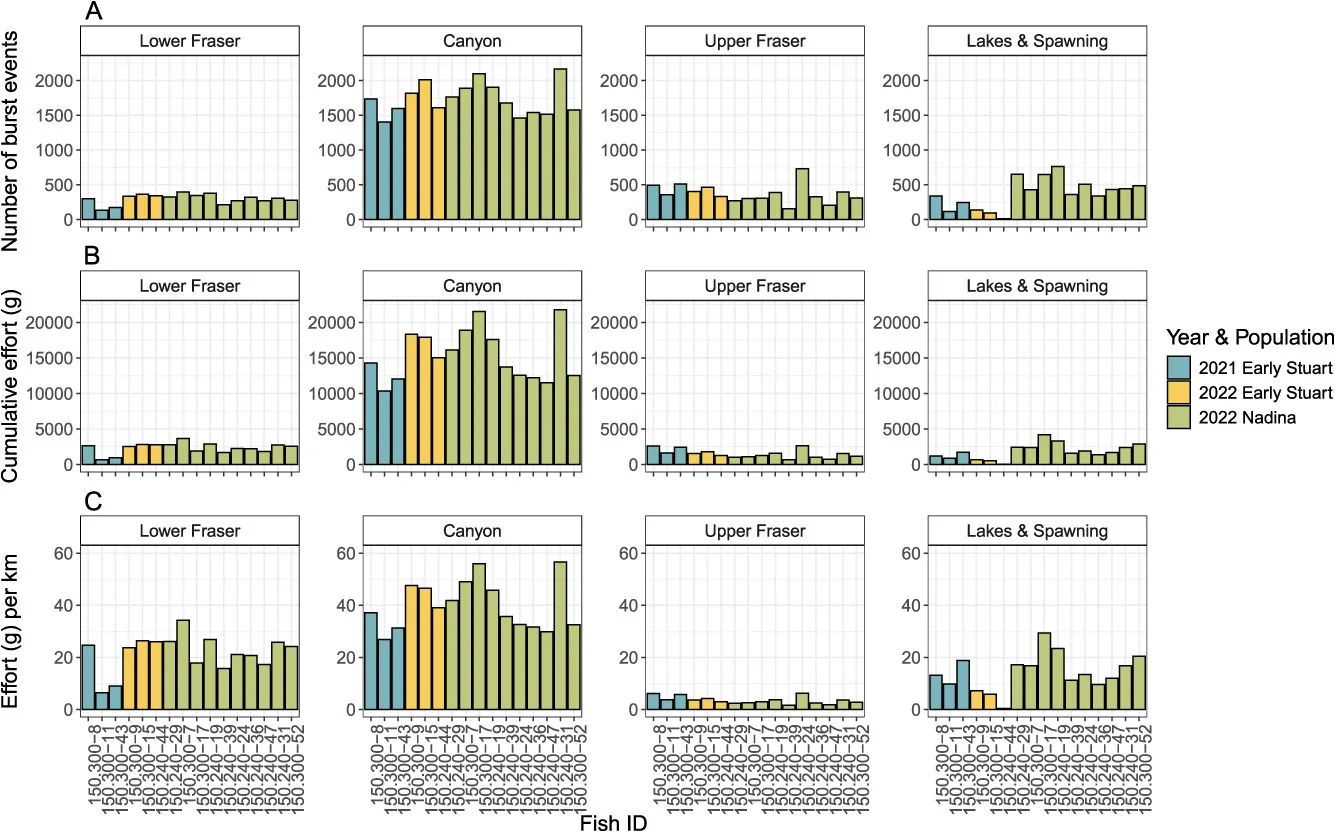
Bursting across river reaches by individual. (A) Number of bursts performed, (B) cumulative burst effort (*g*) and (C) cumulative burst effort per distance unit (*g* km^−1^) per individual at each reach of the Fraser River. Blue and yellow bars represent Early Stuart sockeye salmon tagged in 2021 and 2022, respectively. Green bars represent Nadina sockeye salmon tagged in 2022.

Where, when and how sockeye salmon burst: the tags recorded between 333 749 and 857 353 jerk values (at a 6 s resolution) between time of release and tag recovery on the spawning grounds. Sockeye salmon performed between 2014 and 3437 bursts during their migration, with 1405–2098 bursts occurring in the Fraser Canyon (*∼*63% of all bursts, Fig. 3). On average, sockeye salmon only spent 1.23% of their migration time swimming anaerobically (range: 0.69–1.91%). More specifically, they swam anaerobically for 1.26, 2.44, 0.55 and 0.42% of their time migrating in the Lower Fraser, Fraser Canyon and Upper Fraser and lakes and spawning grounds, respectively. Unsurprisingly, the Fraser Canyon appears to pose a significant challenge (Fig. 3), though to varying degrees among individuals (Fig. 4). Notably, 9 of the 10 Nadina sockeye salmon had at least 1 day with two or fewer bursts around this area (Fig. 3, top panel and Fig. S3). Cumulative bursting effort was an order of magnitude higher in the Fraser Canyon than anywhere else during the migration (Fig. 4), representing an average of 73.5% (range: 65.3–79.4%) of the cumulative bursting effort throughout the migration, despite representing only *∼*35% of the distance. Within each reach, cumulative burst effort per distance was also significantly elevated in the Canyon (40.0 *g* km^−1^, range: 26.9–56.6) and was lowest in the Upper Fraser (3.5 *g* km^−1^, range: 1.6–6.2), with the Lower Fraser (21.6 *g* km^−1^, range: 6.4–34.2) and the lakes and spawning grounds (14.1 *g* km^−1^, range: 0.4–29.4) having intermediate values (Fig. 4).

**Figure 5:**
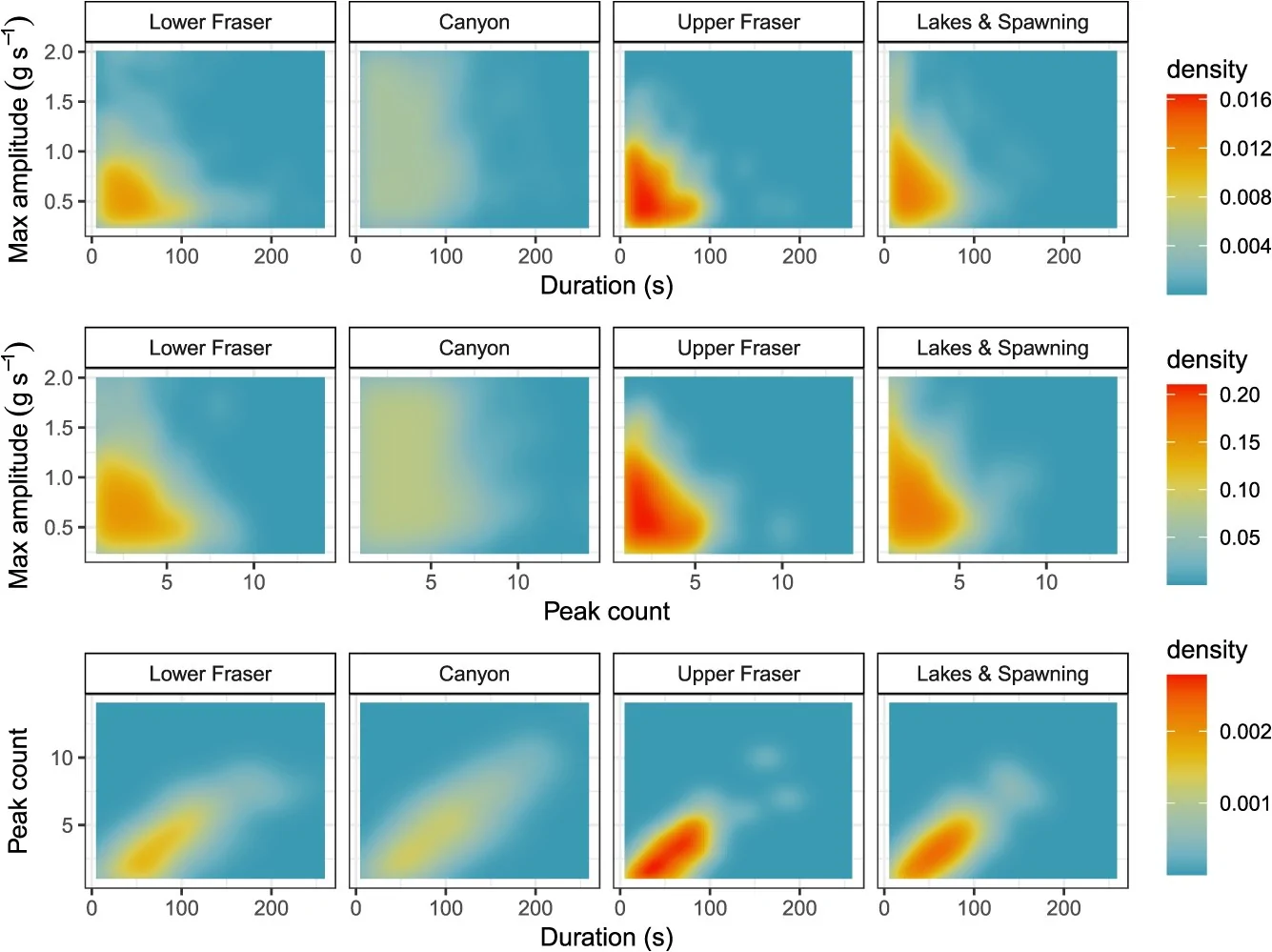
Types of bursts characterized by maximum amplitude (*g* s^−1^), duration (s) and peak count performed in the Lower Fraser, the Fraser Canyon, the Upper Fraser and the lakes and spawning grounds for all individuals across years, emphasizing the markedly different relationship between duration, amplitude and peak count among locations. Note that each panel has its own relative density shading scale (right).

Collectively across all individuals, 43 960 burst events were detected. Of these, 4753 occurred in the Lower Fraser, 27 768 occurred in the Fraser Canyon, 5449 occurred in the Upper Fraser and 5990 occurred in the lakes and spawning creeks. Most bursts were short in duration, low in amplitude and with a single peak (Table 1; Fig. 5), but some were as long as 234 s, with a maximum amplitude of 2.0 *g* s^−1^ (i.e. max out the tag) and with up to 14 peaks (see Fig. S2 e.g. of bursts). Cumulative effort could be as high as 190.92 *g* per event but was on average low (7.61 *g*). Burst characteristics differed across reaches (Table 1). Burst rate, both in terms of number of bursts per kilometre and number of bursts per day, was unsurprisingly highest in the Canyon, but was still relatively high in the lakes and spawning grounds, followed by the Lower Fraser and, lastly, the Upper Fraser (Table 1).

**Table 1:** Summary of burst characteristics in the Lower Fraser, Fraser Canyon, Upper Fraser and the lakes and spawning grounds

**Stretch**	**No. of bursts (per individual)**	**Burst duration (s)**	**Maximum amplitude (*g* s** ^−**1**^ **)**	**No. of peaks**	**Burst effort (*g*)**	**Burst rate**	**Refractory period (min)**
**Lower Fraser (107 km)**	297 (135–398) 10.8%	18.2^a^ (6–234)	0.36^a^ (0.24–2.0)	1^a^ (1–9)	7.79^a^ (0.72–190.92)	2.8 per km 44.8 per day	27.7^a^ (0.6–3218.6)
**Fraser Canyon (385 km)**	1736 (1505–2165) 63.2%	16.6^a^ (6–258)	0.53^b^ (0.24–2.0)	1^a^ (1–14)	8.87^b^ (0.72–178.56)	4.5 per km 117.7 per day	11.7^b^ (0.6–2708.6)
**Upper Fraser (422–425 km)**	341 (156–510) 12.4%	12.0^b^ (6–186)	0.34^a^ (0.24–1.64)	1^b^ (1–10)	4.06^c^ (0.72–91.89)	0.8 per km 33.5 per day	38.1^c^ (0.6–2558.4)
**Lakes and spawning (91–142 km)**	374 (10–761) 13.6%	12.7^b^ (6–168)	0.40^c^ (0.24–2.0)	1^b^ (1–9)	4.87^d^ (0.72–93.24)	3.3 per km 27.0 per day	57.5^d^ (0.6–7102.5)

Presented values are mean values across all sockeye salmon individuals, except for the number of peaks, which shows the median values. The range of values is presented in brackets below. Burst rate is presented in both burst per km and burst per day. Refractory period (minutes) refers to the time between bursts. Here, ‘*g*’ refers to gravity force. Note the distances for the Upper Fraser and lakes and spawning grounds vary slightly between Early Stuart (425, 91 km) and Nadina (422, 142 km). Different letters denote significant differences among reaches within a metric (GLMMs, *P* < 0.05).

Maximum burst amplitude was significantly higher in the canyon than anywhere else along the migration (GLMM, all *P* < 0.0001, Table 1). Maximum burst amplitude was also higher in the lakes and on spawning grounds than in the Lower and Upper Fraser (GLMM, all *P* < 0.0001), but did not differ between Lower and Upper Fraser (GLMM, *P* = 0.999). Burst duration was longer in the Lower Fraser than in the Upper Fraser and in the lakes and spawning grounds (GLMM, all *P*< 0.0001, Table 1), but was similar in the Lower Fraser and in the Canyon (GLMM, *P* = 0.0756). Burst duration was also longer in the Canyon than in the Upper Fraser and in the lakes and spawning grounds (GLMM, all *P* < 0.0001), but did not differ between Upper Fraser and in the lakes and spawning grounds (GLMM, *P* = 0.114). Burst effort differed significantly among all reaches (GLMM, *P**≤* 0.0041, Table 1), but was highest in the Canyon, followed by the Lower Fraser and the lakes and spawning grounds. Burst effort was lowest in the Upper Fraser. Peak count did not differ between the Lower Fraser and the Canyon (GLMM, *P* = 0.970, Table 1), or between the Upper Fraser and the lakes and spawning grounds (GLMM, *P* = 0.757). Peak count however was higher in the Lower Fraser and Canyon than in the Upper Fraser and lakes and spawning grounds (GLMM, all *P* < 0.0001). The time between bursts (refractory period) differed among all reaches, but was shortest in the Canyon, followed by the Lower Fraser, Upper Fraser and the lakes and spawning grounds (GLMM, all *P* *≤* 0.0007, Table 1).

**Figure 6:**
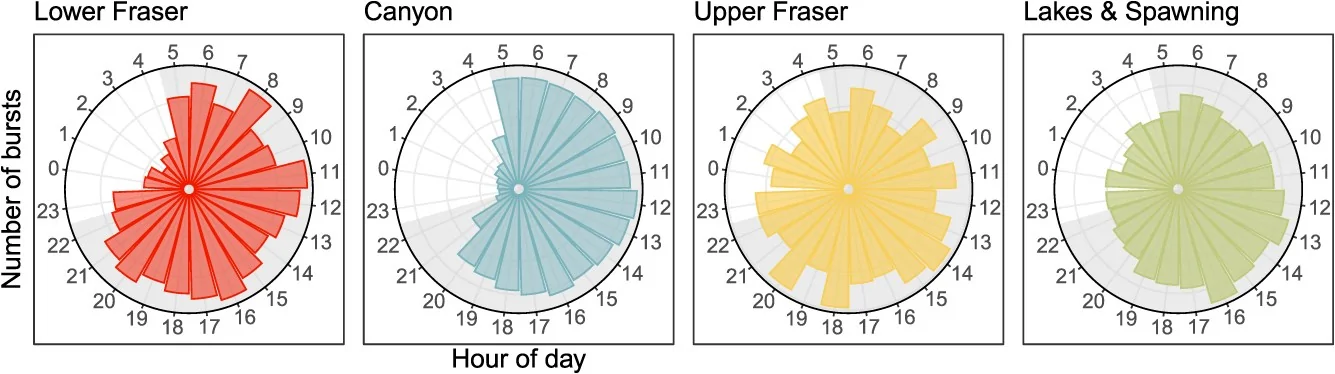
Time of day when bursts were performed in the Lower Fraser (*n* = 4753), the Fraser Canyon (*n* = 27 768), the Upper Fraser (*n* = 5449) and the lakes and spawning grounds (*n* = 5990). Data are pooled for all fish. Note that the size of the bars differs proportionally to each river reach (i.e. bars represent proportionally more data in the canyon as more bursts were performed there). Daytime here is considered as 5:00 to 22:00 as represented by the grey shaded areas.

The vast majority (93.9%) of bursts were performed during daylight hours (here considered to be between 5:00 and 22:00), suggesting a preference for passing difficult areas during daytime. Hour of day had a significant effect on burst count (GLMM, *P* < 0.0001) though this was strongly affected by reach (*P* < 0.0001). More specifically, sockeye salmon appeared to burst almost exclusively during daylight hours in the Lower Fraser and Fraser Canyon, but this pattern is lost once past the Canyon (Fig. 6).

**Figure 7:**
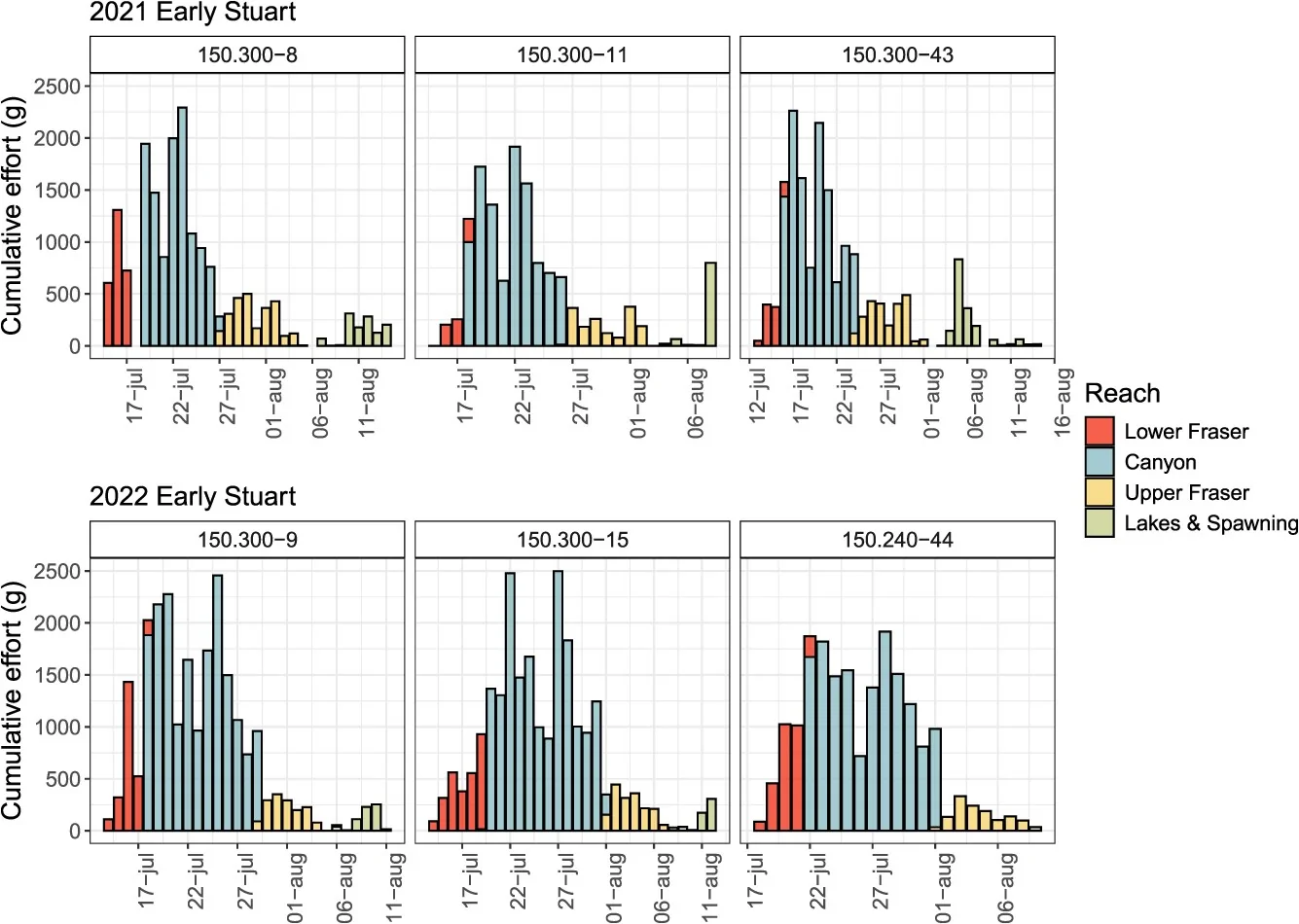
Individual variation in daily cumulative bursting effort across reaches. Cumulative bursting effort (*g*) per day per individual across their full migration for Early Stuart sockeye salmon tagged in 2021 (upper panel) and 2022 (lower panel), emphasizing the total cost of bursting per day and providing a means to compare burst cost through time. Note that the dates on the *x*-axis are only labelled every 5 days.

**Figure 8:**
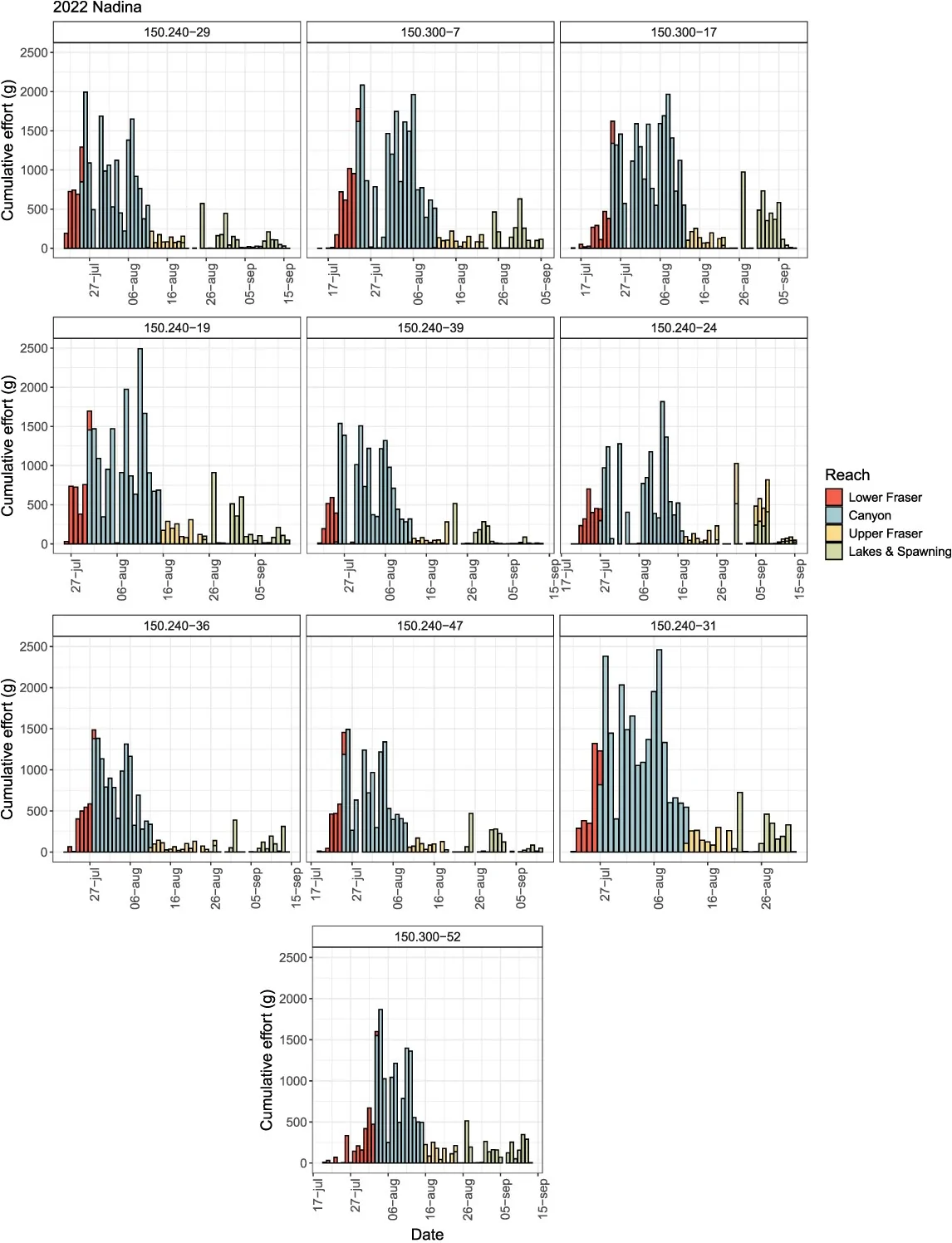
Individual variation in daily cumulative effort across reaches. Cumulative bursting effort (*g*) per day per individual across their full migration for Nadina sockeye salmon tagged in 2022, emphasizing the total cost of bursting per day and providing a means to compare burst cost through time. Note that the dates on the *x*-axis are only labelled every 10 days.

Individual differences: although fish ID was an important factor (i.e. all models retained fish ID as a predictor), interindividual variation was not repeatable. All intraclass correlation coefficients were between 0.001 and 0.003, indicating no specialization among individuals (see Figs S4–S6). That is, all the individuals in this study appeared to perform bursts of similar characteristics. Individuals also showed some variation in their daily cumulative bursting effort (mean = 490 *g*), but no individuals had a higher daily effort than 2802 *g* (Figs 7 and 8). The highest daily cumulative bursting effort occurred in the Canyon for all fish. In fact, the ‘maximum’ daily cumulative bursting effort for each individual ranged between 1383 and 2802 *g*, but averaged at 2059 *g*. Notably, six Nadina sockeye salmon had one or more days with a cumulative bursting effort of zero within the Canyon, but this was never observed in Early Stuart sockeye salmon.

## Discussion

Most Pacific salmon undertake long and difficult once-in-a-lifetime upstream migrations that require both aerobic endurance and anaerobic burst swimming. Qualifying and quantifying those two swimming modes have long been desired by researchers who study Pacific salmon given that a salmon’s fitness is linked to an individual’s capacity for sustained and burst swimming (Brett, 1965; Burnett *et al.*, 2014; Kraskura *et al.*, 2024). Until now, this has not been successfully achieved aside from assessments across short reaches (e.g. Hinch and Rand, 1998; Hinch and Bratty, 2000; Fuchs and Caudill, 2019). However, due to advances in biologging, we now have a clearer understanding of how much effort and intensity sockeye salmon must exert to reach their spawning grounds. This is the first study to quantify and characterize bursting in free-swimming Pacific salmon across the ‘full’ length of the freshwater return migration. We show that sockeye salmon perform thousands of bursts, but only spend ~1.2% of their migration time doing so. This means they spend about 98.8% of their upstream migration either swimming aerobically or resting (our data does not resolve this aspect of swimming). Even in the most difficult part of their migration—the Fraser Canyon—sockeye salmon burst for a relatively small proportion of time (2.4%), and in ways that would suggest energy-conserving behaviour, such as for short durations and with low maximum amplitudes.

Sockeye salmon performed between 2014 and 3437 bursts throughout their migration, with bursting rate and types of bursts performed likely reflecting channel morphology and flow dynamics (Rennie *et al.*, 2018; Venditti *et al.*, 2020; Wright *et al.*, 2022). Sockeye salmon burst most arduously in the Canyon, hydraulically the most challenging reach for salmon, where fish had a bursting rate of 4.5 bursts per km. The longer bursts here were characterized by higher maximum amplitude and greater effort, likely reflecting hydraulic challenges found in the canyon. Interestingly, the bursting rate was also relatively high in the Lower Fraser River (2.8 bursts per km), and bursts were still relatively long, with high maximum amplitudes and high effort. Although the river is nearly 2-km wide at this point, it does have extensive gravel bars that create a lot of variation in the bed topography and thus variation in water depth and velocity. There is also only one main thread of flow, which means there is likely only one narrow swath that is navigable without salmon hitting significant fast and shallow flows. Faced with this, salmon may search for this main thread in a 2 km wide channel or burst intermittently with relatively high effort. It appears that sockeye salmon occasionally choose the latter. Bursting in the Lower Fraser could also reflect the need to escape predators, which are abundant in the Lower Fraser. Bursting rate in the Upper Fraser was lower (0.8 bursts per km), and bursts were of low amplitude, short duration and low effort. Flows in the Upper Fraser are significantly lower, which we expect demands less bursting from fish. Additionally, fatigue and energy constraints may force salmon to burst less powerfully at this stage, conserving energy to complete their migration, continue developing their gonads and secondary sexual characteristics and actively spawn (Williams and Brett, 1987). Finally, the bursting rate was relatively high in the lakes and spawning grounds (3.3 bursts per km). We expect that most of the identified bursts were performed in the spawning creeks as fish were actively spawning, rather than in the lakes. Burst duration, amplitude and effort were lower here than in the Fraser Canyon and Lower Fraser, but higher than in the Upper Fraser, suggesting fish are required to burst with moderate effort on the spawning grounds as they actively spawn (Fuchs and Caudill, 2019). Spawning creeks are characterized by low flow and shallow depths, so moving here indeed requires bursting.

We know that salmon must burst swim to successfully get past many of the areas in the Fraser Canyon (Hinch and Rand, 1998; Hinch and Bratty, 2000). We also know that bursting requires recovery, which has both time and energetic consequences (Birnie-Gauvin *et al.*, 2023). We may expect that longer recovery is necessary following larger, longer bursts, such as in the Fraser Canyon. However, we observed the shortest average refractory period in this reach, likely because salmon need to keep bursting to overcome this challenging river reach. Indeed, these brief refractory periods may represent the shortest possible recovery duration necessary to allow a salmon to resume bursting. In hydraulically easier reaches, we observed longer refractory periods, likely because salmon could afford this time (i.e. they were not immediately faced with another hydraulic challenge), despite bursts being smaller/easier and requiring less recovery in the first place. This is somewhat counterintuitive, but simply aligns with the flow dynamics and channel morphology of the Fraser River. Future work could explore whether a threshold for recovery exists such that individual or cumulative bursts of certain effort require a minimum recovery period.

We observed a shift in bursting temporal patterns during the migration. Sockeye salmon appeared to shift from strict daylight bursting in the Lower Fraser and the Fraser Canyon to bursting at all hours of day in the Upper Fraser (and beyond). Our results clearly showed a preference for bursting and overcoming challenging areas of passage during the daytime, which is commonly observed in salmonids when they navigate difficult conditions (e.g. Kennedy *et al.*, 2013; Nyqvist *et al.*, 2017) presumably because these fish rely on visual cues to navigate turbulent and poor visibility waters (Stuart, 1962; Patterson *et al.*, 2007; Robinson *et al.*, in review). This is supported by the presence of visual predators (seals) in the Lower Fraser that fish must avoid and by sockeye salmon preferentially using the top 1 m of the river during their migration (consistent with visibility based on Secchi depth measurements; Patterson *et al.*, 2007; Robinson *et al.*, in review). However, this trend disappears after the Canyon, likely because flow dynamics are easier to navigate and do not require visual cues, and/or due to an increased urgency to reach spawning grounds (reproductive countdown). Alternatively, these temporal patterns could also provide meaningful benefits for energy conservation as fish continue to develop their gonads during the ascent of the Fraser River.

It appears that sockeye salmon are generally able to minimize their costs and maximize performance during their upstream migration. They enter the river with finite energy reserves that have to be utilized effectively to overcome the Fraser River’s hydraulic challenges, make it to their spawning grounds, develop gonads and successfully spawn. However, it is important to note that this analysis did not include failed migrants which may represent individuals that did not swim in optimal ways. Nonetheless, we found no evidence of individual specialization among successful migrants. Instead, at the scale of this 1100 km migration, it appears that all fish perform similar bursts. When sockeye salmon perform longer bursts, burst amplitude is typically lower. When powerful bursts are necessary, then bursts are typically of short duration and high amplitudes, keeping effort at moderate levels. These patterns suggest fish may modulate burst characteristics in response to conditions, though further research with fine-scale spatial mapping, matched flows and swim speeds are needed to ascertain whether these represent optimal strategies. Individuals did differ slightly in their daily cumulative efforts for bursting (range = 0–2802 *g*, SD = 13.33 *g*), but appeared to maintain overall relatively low effort. A next step in this research will be to characterize bursting in fish that did not successfully make it to their spawning grounds. Do these fish burst too hard too early in their migration? Do we find evidence of different types of bursts in these fish that could help us predict their failure? Some research has found that excessive anaerobic burst activity can lead to delayed mortality (Burnett *et al.*, 2014), so this avenue of research will help determine if excessive bursting is a major cause of en-route mortality, especially in high temperature or high discharge years (Macdonald *et al.*, 2010).

Pacific salmon display species-, population- and sex-specific differences across many characteristics, such as swimming performance (e.g. Kraskura *et al.*, 2020, 2024), thermal performance and tolerance (e.g. Eliason *et al.*, 2011; Van Wert *et al.*, 2023; Mayer *et al.*, 2024) and behaviour (e.g. Burnett *et al.*, 2014; Eliason *et al.*, 2020). As such, we expected to observe differences in bursting characteristics between Early Stuart sockeye salmon and Nadina sockeye salmon, as well as between males and females. Unfortunately, given our low sample sizes (sample size of one to five per group), we were unable to make these comparisons. However, we did find some evidence that Nadina and Early Stuart sockeye salmon differ in their bursting behaviour: nine of the ten Nadina fish had at least 1 day with two or less bursts while in the Canyon. In contrast, none of the six Early Stuart sockeye salmon displayed this potential resting behaviour. There are a few potential drivers of these behaviour differences. Early Stuart fish may have superior bursting capacity, incurring lower metabolic costs from burst swimming. As a result, Early Stuart sockeye salmon might have lower recovery costs or require less recovery time, allowing them to repeat burst swims more frequently. We have no evidence suggesting Early Stuart sockeye salmon incur lower costs however, so these differences in bursting tendencies may instead reflect the urgency to reach spawning grounds. Early Stuart fish are on a constrained migration timeline and must make it to the spawning grounds more quickly and thus may be more motivated to continuously migrate without extended breaks. Nadina sockeye salmon spawn later despite beginning their migration at a similar time and thus can afford to wait and burst differently. Given the few recovered tags from females, we are unable to draw any conclusions related to sex-specific differences in bursting at this stage, though future work should explore this avenue, especially considering the higher en-route mortality for female Pacific salmon (Hinch *et al.*, 2021).

Swimming performance is fundamental to how and why salmon undergo successful migration or not. Salmon engage in various types of swimming modes as they migrate upstream (Jahn and Seebacher, 2022; Wilson *et al.*, 2022), from the steady sustained swimming, which does not lead to fatigue, to burst-and-coast swimming, which eventually leads to fatigue, and finally bursting, which is unsustainable over long periods and quickly leads to fatigue (Brett *et al.*, 1958; Kieffer, 2000; Castro-Santos, 2005; Holder *et al.*, 2022). Understanding the quantity and type of bursting that sockeye salmon perform can help us identify areas that are particularly challenging for fish and assess the causes of mortality along their migration. This is critical because repeated anaerobic activity prolongs recovery which is detrimental to fish because it is energetically costly, delays migration and impairs predator-avoidance behaviours (Eliason and Farrell, 2016; Birnie-Gauvin *et al.*, 2023). The true cost of bursting—including (i) the actual ATP generated by less efficient glycolysis instead of oxidative phosphorylation, (ii) the excess post-exercise oxygen consumption, and (iii) the recovery costs to regenerate high energy phosphate stores and restoring ion, acid–base and hormone balance—is however unknown for large, adult salmon and represents a major research gap (Birnie-Gauvin *et al.*, 2023). Here, perhaps unsurprisingly, we identified the Fraser Canyon as particularly challenging, where cumulative effort was an order of magnitude higher than anywhere else in the Fraser River. Our data can be used to infer specific areas of concern by identifying river sections with the highest bursting rate or cumulative bursting effort and where salmon are most likely to fail. A natural next step for this research would be to characterize the energetic costs of migration on a fine-scale basis through environmental (flow and morphology) mapping (Wright *et al.*, 2024) and bioenergetic modelling, as well as assessing bursting in unsuccessful fish. We know how much energy fish have at the start and end of their migration (Idler and Clemens, 1959), but where this energy is specifically used and how that varies with flow and temperature are largely unknown (Patterson *et al.*, 2004). Such information can be used by resource managers and conservation decision-makers to identify opportunities for habitat protection or enhancement. In a warming world or in the face of future landslides, active habitat management may be needed to ensure that Pacific salmon are able to reach spawning grounds without encountering conditions that exceed their bursting capacity or result in somatic energetic depletion such that they are unable to reproduce. Since our focus was on the full migration, identifying these areas was outside the scope of this paper. However, applying these electronic tagging tools could significantly enhance conservation efforts and management practices at specific reaches of interest.

## Supplementary Material

Web_Material_coag066

## Data Availability

Data will be made available upon request.
