# Supplementary material for: Burst swimming in sockeye salmon during their 1100 km upstream migration
Source: Conserv Physiol. 2026 Sep 12;14(1):coag066. doi: 10.1093/conphys/coag066 (PMC13570124; doi:10.1093/conphys/coag066)
Supplement: Supplementary file 1 [file web_material_coag066.docx]

**Supplementary materials**

***Section 1: Supplementary figures***

*Figure S1*. Discharge (m s^-3^, blue) and temperature (red) in the Lower Fraser River between June and September 2021 and 2022. The shaded grey area represents the period during which we have jerk data for sockeye salmon in this study (12 July to 16 September).

*Figure S2*. Examples of representative bursts. Burst 238 is long, but has a low maximum amplitude, and 7 peaks, exemplifying a long but low effort burst. Burst 1284 is similar but with higher amplitudes at each peak. Both burst 238 and burst 1284 *could* be representative of burst-and-coast swimming. Burst 292 and 1127 have 3 and 2 peaks respectively, but a higher maximum amplitude, exemplifying powerful bursts, likely necessary to overcome hydraulic challenges.

*Figure S3 (panel A)*. Raw jerk (*g*·s^-1^) and number of burst events per day during the migration of sockeye salmon, coloured by river reach. The dashed horizontal line at 0.24 indicates the bursting threshold. Note that dates are only labelled every 10 days. Year and population are indicated in brackets after fish ID.

*Figure S3 (panel B)*. Raw jerk (*g*·s^-1^) and number of burst events per day during the migration of sockeye salmon, coloured by river reach. The dashed horizontal line at 0.24 indicates the bursting threshold. Note that dates are only labelled every 10 days. Year and population are indicated in brackets after fish ID.

*Figure S3 (panel C)*. Raw jerk (*g*·s^-1^) and number of burst events per day during the migration of sockeye salmon, coloured by river reach. The dashed horizontal line at 0.24 indicates the bursting threshold. Note that dates are only labelled every 10 days. Year and population are indicated in brackets after fish ID.

*Figure S3 (panel D)*. Raw jerk (*g*·s^-1^) and number of burst events per day during the migration of sockeye salmon, coloured by river reach. The dashed horizontal line at 0.24 indicates the bursting threshold. Note that dates are only labelled every 10 days. Year and population are indicated in brackets after fish ID.

*Figure S3 (panel E)*. Raw jerk (*g*·s^-1^) and number of burst events per day during the migration of sockeye salmon, coloured by river reach. The dashed horizontal line at 0.24 indicates the bursting threshold. Note that dates are only labelled every 10 days. Year and population are indicated in brackets after fish ID.

*Figure S3 (panel F)*. Raw jerk (*g*·s^-1^) and number of burst events per day during the migration of sockeye salmon, coloured by river reach. The dashed horizontal line at 0.24 indicates the bursting threshold. Note that dates are only labelled every 10 days. Year and population are indicated in brackets after fish ID.

*Figure S3 (panel G)*. Raw jerk (*g*·s^-1^) and number of burst events per day during the migration of sockeye salmon, coloured by river reach. The dashed horizontal line at 0.24 indicates the bursting threshold. Note that dates are only labelled every 10 days. Year and population are indicated in brackets after fish ID.

*Figure S3 (panel H)*. Raw jerk (*g*·s^-1^) and number of burst events per day during the migration of sockeye salmon, coloured by river reach. The dashed horizontal line at 0.24 indicates the bursting threshold. Note that dates are only labelled every 10 days. Year and population are indicated in brackets after fish ID.

*Figure S4*. Types of bursts characterized by maximum burst amplitude (g · s^-1^) and duration (s) for all individuals across the full migration. Year of tagging and population are indicated in the brackets.

*Figure S5*. Types of bursts characterized by peak count and duration (s) for all individuals across the full migration. Year of tagging and population are indicated in the brackets.

*Figure S6*. Types of bursts characterized by maximum burst amplitude (g · s^-1^) and peak count for all individuals across the full migration. Year of tagging and population are indicated in the brackets.

## ***Section 2: Calibration study for jerk tag***

The goal of this calibration was to match logged jerk values to a threshold between aerobic or anerobic swim modes. To establish the transition between these modes, we first evaluated the relationship between jerk and tailbeat frequency (TBF) using triaxial accelerometers. We then related TBF to swim speed using published work on the transition from aerobic to anaerobic fueling to ultimately establish a jerk threshold for burst swimming.

To collect paired jerk and TBF data, we conducted swim trials on adult sockeye salmon using a 10m long high-velocity flume (Venditti et al. 2016) at Simon Fraser University, British Columbia, Canada (Figure S7). Temperatures in the flume were between 17 and 18°C, and within the range of temperatures sockeye salmon experience during their migration in the Fraser River. Four male study fish (fork length: mean 62.1 cm, range 59.9–64.5 cm) were tagged with both a gastrically inserted jerk tag and an externally attached accelerometer (Axy5S; TechnoSmart, Rome, Italy). The gastric insertion of the jerk tag mimicked the application protocols used in the study presented here. The external attachment of the accelerometer allowed us to collect high resolution triaxial acceleration data to calculate TBF and relate to jerk values. Two trials were completed per fish, where individuals were placed in the flume and the velocity was progressively increased up to a maximum of 2.7 m s^-1^. The swim trials were video recorded, providing supplemental information on swim behaviour (e.g., forward movement).

Figure S7. Double-tagged sockeye salmon in 10 m long flume during the calibration study.

The jerk tags used in the main study record the average rate of change in acceleration over a 6 s sampling window. Jerk acceleration is calculated from three axis, internally sampled at 50 Hz, and summed using the following equation:

This results in four values per internal sample that are individually averaged over the sampling window to produce the logged dataset presented here. The scaling factor adjusts the summed triaxial acceleration into discrete levels of change in absolute acceleration, scaled from 0 to 2 *g*·s^-1^, with a resolution of 0.04 *g*·s^-1^. For this calibration study, two sockeye were tagged with 6 s jerk tags, and two sockeye were tagged with 2 s tags. We included the 2 s sampling window to evaluate the sensitivity of a shorter mean logging interval on the relationship with TBF and ultimately swim speed estimation.

TBF and maximum absolute relative tailbeat amplitude (TBA) were extracted from dynamic acceleration in the y-axis using the same 2 or 6 s sampling window as the paired jerk tag (Figure S8, panel A; Shepard at el. 2008; Warren-Myers et al. 2023). TBF was regressed against jerk to evaluate the relation between these two measures for both sampling durations (Figure S8, panel B). The relation between TBF and jerk for the two sampling durations were similar, allowing us to combine the data sets in subsequent steps.

*Figure S8*. Tailbeat frequency (TBF) in relation to maximum absolute tailbeat amplitude (TBA; top) and mean jerk values (bottom) from tags with 2s (black) and 6 s (red) sampling windows. Linear regressions were fit to each TBF and jerk sampling window duration to visualize the role of sampling window.

We assessed the relationship between maximum relative TBA and TBF to determine when maximum relative TBA starts to increase (Figure S8, panel A). Amplitude increases with swimming speed as a power function. Under slow, steady-state aerobic swimming, velocity increases are powered by an increase in TBF and amplitude remains relatively constant. At higher speeds, when salmon transition to unsteady swimming, TBA increases (Bainbridge 1958; Webb 1971, 1975). Notably, jerk and maximum TBA display similar patterns with TBF. Figure S8 indicates that amplitude is consistent until a TBF of ~3.5-4 Hz.

To partition jerk data into anaerobic burst swim events using TBF, we first identified two body lengths (BL) per second as the relative swim speed transition from aerobic to anaerobic fueling in salmonids (Kraskura et al. 2024). Salmonids transition from steady-state aerobic swimming to anaerobic burst-and-coast swimming between 70-80% of their critical swimming velocity (Ucrit), as identified using EMG tags and lactate and phosphocreatine levels in the blood (Burgetz et al. 1998; Geist et al. 2003). Adult Early Stuart sockeye salmon Ucrit is reported to be 2.4–2.7 BL s^-1^ (MacNutt et al. 2006; Eliason et al. 2013), thus 70–80% of Ucrit is ~2 BL s^-1^ for Early Stuart fish.

We then used the following swim speed equation published in Bainbridge (1958) to calculate the tailbeat frequency that equates to 2 BL s^-1^:

Where is the swim speed in cm s^-1^, the tailbeat frequency in beats per second, and the body length in cm. A relative swim speed of 2 BL/s equates to an absolute swim speed of approximately 124 cm s^-1^ herein. Thus, we estimate an anaerobic TBF threshold of 4 Hz based on the average body length of study fish used in this calibration experiment.

The relationship between TBF and jerk during forward movement within the swim flume was evaluated for both sampling windows (Figure S9). We selected forward movement because we were most interested in the intensity relationship between TBF and jerk during the transition to burst propulsion events. Note however that even when including all swim categories (backwards motion, stationary swimming and forward movement), the jerk threshold is the same. There was a significant positive linear relationship between TBF and jerk, with an R^2^ of 0.70 (slope = 0.22, intercept = -0.68). Using the linear equation, we predicted a jerk bursting threshold of 0.2 *g*·s^-1^ (95% PI: 0.16–0.25 *g*·s^-1^) when fish are swimming at 4 Hz. This is consistent with the 0.21 *g*·s^-1^ jerk threshold that Fuchs and Caudill (2019) used as an upper limit for differentiating holding behaviour of reproductive steelhead trout from more active spawning behaviours (e.g. digging, oviposition, aggression, burst movement) using the same tag model (4-s sampling window). For this paper, we set the jerk threshold for burst swimming at 0.24 *g*·s^-1^, one jerk level above our predicted value, to limit possible false burst events.

*Figure S9*. The relationship between TBF and jerk collected during forward swimming movement during the swim flume trials. The line and associated shading depicts the linear relationship with 95% confidence interval.

Our jerk threshold selection was also supported by the jerk data recorded in our data set at migration areas known to be less physically demanding, where we would expect salmon to be using sustained aerobic swimming. In these areas, jerk values were typically maintained between 0 and 0.20 *g*·s^-1^ (inclusively), but rarely surpassing our threshold of 0.24 *g*·s^-1^*.*

We further conducted a simple sensitivity assessment of the threshold value to evaluate the influence of a small shift in value on the outcomes. Lowering the threshold to 0.20 *g*·s^-1^ of course resulted in more bursts throughout the migration because the threshold of 0.20 *g·s^-1^* was surpassed more frequently, including in areas known not to be challenging for salmon. Bursts were of course also of slightly longer duration. However, the outcomes of the analyses did not change. The differences in burst types between the reaches remain unchanged. Given this, we feel confident that using a threshold of 0.24 *g*·s^-1^ does not overestimate bursting during sockeye salmon migration.

**References**

Bainbridge, R. (1958). The Speed of Swimming of Fish as Related to Size and to the Frequency and Amplitude of the Tail Beat. Journal of Experimental Biology, 35(1), 109–133.

Burgetz, I.J., Rojas-Vargas, A., Hinch, S.G. & Randall, D.J. (1998). Initial recruitment of anaerobic metabolism during sub-maximal swimming in rainbow trout (*Oncorhynchus mykiss*). Journal of Experimental Biology, 201(19), 2711–2721.

Eliason, E.J., Clark, T.D., Hinch, S.G. & Farrell, A.P. (2013). Cardiorespiratory performance and blood chemistry during swimming and recovery in three populations of elite swimmers: adult sockeye salmon. Comparative Biochemistry and Physiology Part A: Molecular & Integrative Physiology, 166(2), 385–397.

Fuchs, N.T. & Caudill, C.C. (2019). Classifying and inferring behaviors using real‐time acceleration biotelemetry in reproductive steelhead trout (*Oncorhynchus mykiss*). Ecology and Evolution, 9(19), 11329–11343.

Geist, D.R., Brown, R.S., Cullinan, V.I., Mesa, M.G., Vanderkooi, S.P. & McKinstry, C.A. (2003). Relationships between metabolic rate, muscle electromyograms and swim performance of adult chinook salmon. Journal of Fish Biology, 63(4), 970–989.

Kraskura, K., Patterson, D.A. and Eliason, E.J. (2024). A review of adult salmon maximum swim performance. Canadian Journal of Fisheries and Aquatic Sciences, 81(9), 1174–1216.

MacNutt, M.J., Hinch, S.G., Lee, C.G., Phibbs, J.R., Lotto, A.G., Healey, M.C. & Farrell, A.P. (2006). Temperature effects on swimming performance, energetics, and aerobic capacities of mature adult pink salmon (*Oncorhynchus gorbuscha*) compared with those of sockeye salmon (*Oncorhynchus nerka*). Canadian Journal of Zoology, 84(1), 88–97.

Shepard, E., Wilson, R., Halsey, L., Quintana, F., Gómez Laich, A., Gleiss, A., Liebsch, N., Myers, A. & Norman, B. (2008). Derivation of body motion via appropriate smoothing of acceleration data. Aquatic Biology, 4, 235–241.

Venditti, J. G., Lin, C. Y. M., & Kazemi, M. (2016). Variability in bedform morphology and kinematics with transport stage. Sedimentology, 63(4), 1017-1040.

Warren-Myers, F., Svendsen, E., Føre, M., Folkedal, O., Oppedal, F. & Hvas, M. (2023). Novel tag-based method for measuring tailbeat frequency and variations in amplitude in fish. Animal Biotelemetry, 11(1), 12.

Webb, P. W. (1971). The swimming energetics of trout: I. Thrust and power output at cruising speeds. Journal of Experimental Biology, 55(2), 489–520.

Webb, P. W. (1975). Acceleration performance of rainbow trout *Salmo gairdneri* and green sunfish *Lepomis cyanellus*. Journal of Experimental Biology, 63(2), 451–465.
